# Rapid fluorometric detection of drug resistant tumour cells.

**DOI:** 10.1038/bjc.1985.238

**Published:** 1985-10

**Authors:** A. deFazio, M. H. Tattersall


*Br. J. Cancer* (1985), **52**, 633–636

### Short Communication

### Rapid fluorometric detection of drug resistant tumour cells

A. deFazio & M.H.N. Tattersall

*Ludwig Institute for Cancer Research (Sydney Branch), Blackburn Building, University of Sydney, Sydney, NSW 2006, Australia*

A rapid estimation of mutation frequency in tumours would be invaluable in cancer management. Considerable evidence suggests that drug resistance in tumours frequently arises as a consequence of spontaneous somatic mutation (Goldie & Coldman, 1979) and therefore the mutation rate of a tumour will be an index of the potential to develop drug resistance. Previous studies have compared the mutation rates of cell lines using cloning assays. Cifone & Fidler (1981) reported a higher mutation rate in metastatic variant of UV-2237 fibrosarcoma cells than in a clone of the same cell line with lower metastatic potential. Warren *et al.* (1981) reported that fibroblasts from patients with Bloom syndrome, which predisposes individuals to various cancers, had a 10–15 fold higher mutation rate than did fibroblasts from normal individuals. These findings have been important in correlating malignant capacity with genetic instability, but unfortunately, the techniques used have limited general application since few human tumour cells will form colonies on plastic. An alternative assay, soft agar cloning, has also been used for the determination of mutation rates in a variety of mammalian cell lines, including Chinese hamster ovary cells (Li & Shimizu, 1983) and the L5178Y mouse lymphoma cell line (Irr & Snee, 1982). Again, the low cloning efficiency of human tumour cells in soft agar (Hamburger *et al.*, 1978) and the fact that this method selects only mutant cells which clone in agar limits the use of this assay to determine mutation frequency in human tumours. This latter source of error might bias the estimation of the mutation rate in favour of more malignant cells, which generally have higher cloning efficiencies (Elmore *et al.*, 1983).

Morley *et al.* (1983) and Albertini *et al.* (1982) have reported a limiting dilution technique for the measurement of mutation frequency in human lymphocytes. Culture conditions have been optimised for peripheral blood lymphocytes and a cloning efficiency of 20–60% has been obtained. Problems have been encountered, however, in studies of cultured lymphoblast lines with variable cloning efficiencies and different growth factor requirements (Seshadri *et al.*, 1984).

We have prepared a monoclonal antibody to bromodeoxyuridine (BrUdR) which recognises BrUdR-substituted DNA. BrUdR is a thymidine analogue readily incorporated into DNA by proliferating cells. Cells which incorporate BrUdR after exposure to a lethal concentration of a cytotoxic drug must be drug resistant and may be identified as such by the monoclonal antibody using immunofluorescence techniques. If the drug used is a selective agent for cells with a specific somatic mutation e.g. the purine analogue 6-thioguanine (6-TG) which selects for cells with a mutation at the hypoxanthine- guanine phosphoribosyltransferase (HGPRT) locus, then a measure of mutant frequency is obtained.

The monoclonal antibody to BrUdR was derived from the fusion of SP2/0-Ag14 cells (Shulman *et al.*, 1978) to spleen cells from BALB/c mice immunized with bromouridine conjugated to bovine serum albumin (BSA) (Köhler & Milstein, 1975; Erlanger & Beiser, 1964). The antibody with highest affinity and specificity for BrUdR in the DNA strand was chosen using a competitive enzyme linked immuno-sorbent assay (ELISA) (Voller *et al.*, 1978). Although this antibody identifies cells incorporating BrUdR after only minutes of exposure, an incubation period equivalent to one cell cycle time ensures that all cycling cells will have traversed S-phase and thus incorporated BrUdR into DNA. A fluorescence histogram of cells exposed to BrUdR for one cell cycle time is shown in Figure 1. Superimposed on this histogram of a highly fluorescent cell population is the background fluorescence peak obtained from stained cells which had not been exposed to BrUdR. This fluorescence is identical to the autofluorescence produced by unstained cells (not shown), indicating negligible non-specific binding of the anti-BrUdR antibody.

BrUdR incorporation and subsequent fluorescent staining, was used to investigate the 6-TG dose response relationships flow cytometrically. The dose reponse curves illustrated in Figure 2 show that for CCRF-CEM cells a concentration of over 30 $\mu$M 6-TG for 72 h is necessary to stop DNA synthesis completely in 'wild type' cells. The dose-response

Correspondence: A. deFazio
Received 12 February 1985.

© The Macmillan Press Ltd., 1985

**Figure 1** Immunofluorescent staining of proliferating cells exposed to BrUdR. CCRF-CEM cells, a human leukaemia T-cell line, growing exponentially in RPMI-1640 medium supplemented with glutamine and 10% foetal calf serum, were exposed to $10^{-5}$ M BrUdR for 24 h (equivalent to one cell cycle). This exposure was shown to have no affect on the growth rate or cell cycle phase distribution of these cells. Cells ($2 \times 10^6$) were washed, resuspended in 5 ml of PBS and slowly syringed into 15 ml of cold, vortexing ethanol. The fixed cell suspensions were kept at 4°C until staining. The fixed cells were pelleted and the DNA denatured *in situ* by resuspension in 1 ml of 1.5 M HCl. After 20 min at room temperature the cells were washed in cold saline/1% Tween 20 (polyoxyethylene sorbitan monolaurate, Sigma, St Louis, MO, USA) and incubated for 30 min at 37°C with protein A purified anti-BrUdR antibody 200 μg ml$^{-1}$, was made up in PBS/1% BAS/1% Tween 20. The cells were washed in this buffer and incubated with sheep F(ab′)2 anti-mouse Ig-fluorescein conjugated (from New England Nuclear, Boston, Massachusetts) 100 μl at 250 μg ml$^{-1}$, for 60 min at 37°C. After a final wash the cells were resuspended in buffer and the fluorescence measured using a fluorescence-activated cell sorter (FACS III, Becton Dickinson, Sunnyvale, Ca, USA). The laser was tuned to 488 nm at 400 mW. ▩ represents control fluorescence from staining cells not exposed to BrUdR.

**Figure 2** 6-thioguanine dose-response curves. Exponentially growing CCRF-CEM cells were exposed to varying concentrations of 6-TG for 48 h (▲) and 72 h (●). $10^{-5}$ M BrUdR was added to each flask 24 h prior to harvesting. This exposure to BrUdR had no effect on the growth of control cells, nor did it affect the response of drug-treated cells. The cells were washed, fixed and stained for BrUdR incorporation as described in the legend of **Figure 1.** The surviving fraction was determined from the proportion of fluorescent cells as analysed using the FACS III. The total number of cells in each sample and the fluorescent population were determined by the simultaneous measurement of forward light scatter and fluorescence.

relationship detected by this assay is in close agreement with the results for peripheral blood lymphocytes and human skin fibroblast lines reported by others using limiting dilution and cloning assays, respectively (Albertini *et al.*, 1982; Elmore *et al.*, 1983). Using this information, conditions chosen for the detection of resistant cells were 72 h exposure to 100 μM 6-TG. This treatment is toxic to wild-type CCRF-CEM cells, but the growth rate of a mutant CCRF-CEM sub-line, HGPRT-1 (Waddell & Ullman, 1983) which is deficient in the enzyme HGPRT, is identical to that of untreated control cells under these conditions (data not shown).

Figure 3 illustrates fluorescence historgrams of mutant, wild type and mixed cell populations exposed to 6TG. Histogram (A) shows that the HGPRT-1 mutants continue synthesizing DNA normally in the presence of a high concentration of 6-TG which stops DNA synthesis in the wild-type CCRF-CEM cells (B). In the latter case there is no fluorescence peak indicating that the cells are not incorporating BrUdR. Mixtures of mutants and wild type CCRF-CEM cells in varying proportions were exposed to 6-TG in order to investigate the sensitivity of the BrUdR assay in the rapid detection of low numbers of drug-resistant cells. The histogram (C) is obtained from a mixture of one HGPRT-1 mutant in $10^5$ wild-type CCRF-CEM cells. A small but easily distinguishable peak of fluorescent cells was obtained, representing

**Figure 3** Fluorescence histograms obtained from a) HGPRT-1, a mutant subline of CCRF-CEM, b) the 'wild type' CCRF-CEM cells and c) a mixture of 1 mutant in $10^5$ non-mutant CCRF-CEM cells after exposure to $10^{-4}$M 6-TG for 72h with $10^{-5}$M BrUdR added for the final 24h. The total number of exponentially growing cells in each flask at the time of 6-TG addition was $1.7 \times 10^7$. The HGPRT-1 cells (a) continued to cycle normally and at 72h, $2 \times 10^6$ cells were washed, fixed and stained for BrUdR incorporation, as described in the legend to **Figure 1.** The entire contents of flasks depicted in b and c, were harvested and stained, as substantial cell death had decreased the total number of whole cells (as determined by haemocytometer count) to $<2 \times 10^6$. The mutant cell population in c, has clearly expanded during this period and although accurate quantitation is not yet possible at least 800 fluorescent cells are detected in the histogram shown.

cycling mutant cells. The possibility of metabolic co-operation between the resistant and sensitive cells (Ochi *et al.,* 1982) in mix experiments was examined but no evidence of this phenomenon was found. Accurate quantitation of rare cell sub-populations such as the HGPRT-cells in a mix experiment is being attempted using fluorescent beads to determine sample volume and thus cell number (Stewart & Steinkamp, 1982).

The BrUdR antibody method of identifying drug-resistant cells has many potential advantages. The method does not require high cloning efficiency conditions and provided adequate concentrations of 6-TG are used for a sufficient exposure period to kill sensitive cells, cells continuing to cycle can be assumed to be HGPRT-. As in the autoradiographic method for detection of 6-TG resistant lymphocytes described by Strauss and Albertini (1979), cells identified as 6-TG resistant by BrUdR fluorescence cannot be proved to be mutants since they cannot be clonally expanded and the HGPRT activity measured. However Dempsey and Morley (1983) have shown, in parallel cloning and autoradiographic mutation assays in peripheral blood lymphocytes, that provided a 6-TG concentration well along the plateau of the dose-response curve is used, resistant cells measured autoradiographically usually completely lack HGPRT.

This rapid and sensitive assay can be applied in a wide range of cells. It will find an important place not only as a drug resistance assay, measuring the proportion of cells that are resistant to a chemotherapeutic agent, but also as a mutation assay measuring the rates with which tumour cells acquire drug resistance. Clearly the precise conditions for studying drug resistance in human tumours remain to the defined and currently we are using this reagent to study biopsies of xenografted tumours. The duration of drug exposure *in vitro* and the timing of BrUdR addition may need to be varied depending upon the cytotoxic drug being tested and the growth fraction of the tumour. A similar antibody (Gratzner, 1982) has recently been used to measure the proliferating cell fraction *in vivo* (Morstyn *et al.,* 1983) and to study cell cycle perturbations (Dolbeare *et al.,* 1983).

We are grateful to Elizabeth A. Musgrove for her advice and technical expertise in flow cytometry, and to Judy Hood for typing this manuscript.

### References

ALBERTINI, R.J., CASTLE, K.L. & BORCHERDING, W.R. (1982). T-cell cloning to detect the mutant 6-thioguanine-resistant lymphocytes present in human peripheral blood. *Proc. Natl Acad. Sci.* (*USA*), **79**, 6617.

CIFONE, M.A. & FIDLER, I.J. (1981). Increasing metastatic potential is associated with increasing genetic instability of clones isolated from murine neoplasms. *Proc. Natl Acad. Sci.* (*USA*), **78**, 6949.

DEMPSEY, J.L. & MORLEY, A.A. (1983). Evidence that thioguanine-resistant lymphocytes detected by autoradiography are mutant cells. *Mutat. Res.*, **119**, 203.

DOLBEARE, F., GRATZNER, H., PALLAVICINI, M.G. & GRAY, J.W. (1983). Flow cytometric measurement of total DNA content and incorporated bromodeoxyuridine. *Proc. Natl Acad. Sci.* (*USA*), **80**, 5573.

ELMORE, E., KAKUNAGA, T. & BARRETT, J.C. (1983). Comparison of spontaneous mutation rates of normal and chemically transformed human skin fibroblasts. *Cancer Res.*, **43**, 1650.

ERLANGER, B.F. & BEISER, S.M. (1964). Antibodies specific for ribonucleosides and ribonucleotides and their reaction with DNA. *Proc. Natl Acad. Sci.* (*USA*), **52**, 68.

GOLDIE, J.H. & COLDMAN, A.J. (1979). A mathematic model for relating the drug sensitivity of tumors to their spontaneous mutation rate. *Cancer Treat. Rep.*, **63**, 1727.

GRATZNER, H.G. (1982). Monoclonal antibody to 5-bromo- and 5-iododeoxyuridine: A new reagent for detection of DNA replication. *Science*, **218**, 474.

HAMBURGER, A. W., SALMON, S.E., KIM, M.B. & 4 others. (1978). Direct cloning of human ovarian carcinoma cells in agar. *Cancer Res.*, **38**, 3438.

IRR, J.D. & SNEE, R.D. (1982). A statistical method for analysis of mouse lymphoma L5178Y cell TK locus forward mutation assay. *Mutat. Res.*, **97**, 371.

KÖHLER, G. & MILSTEIN, C. (1975). Continuous cultures of fused cells secreting antibody of predefined specificity. *Nature*, **256**, 495.

LI, A.P. & SHIMIZU, R.W. (1983). A modified agar assay for the quantitation of mutation at the hypoxanthine guanine phosphoribosyl transferase gene locus in Chinese hamster ovary cells. *Mutat. Res.*, **111**, 365.

MORLEY, A.A., TRAINOR, K.J., SESHADRI, R. & RYALL, R.G. (1983). Measurement of *in vivo* mutations in human lymphocytes. *Nature*, **302**, 155.

MORSTYN, G., HSU, S-M., KINSELLA, T., GRATZNER, H., RUSSO, A. & MITCHELL, J.B. (1983). Bromodeoxyuridine in tumors and chromosomes detected with a monoclonal antibody. *J. Clin. Invest.*, **72**, 1844.

OCHI, T., OHSAWA, M., NODA, K. & UMEDA, M. (1982). Improvement in recovery of 6-thioguanine-resistant mutants by the use of inhibitors of metabolic cooperation in *in vitro* mutagensis assay. *Jpn J. Exp. Med.*, **52**, 291.

SESHADRI, R., MATTHEWS, C., GARDIAKOS, C. & MORLEY, A.A. (1984). The effect of bone marrow stromal cells on the cloning of human leukaemia/lymphoma cell lines. *Int. J. Cell. Cloning*, (in press).

SHULMAN, M., WILDE, C.D. & KÖHLER, G. (1978). A better cell line for making hybridomas secreting specific antibodies. *Nature*, **276**, 269.

STEWART, C.C. & STEINKAMP, J.A. (1982). Quantitation of cell concentration using the flow cytometer. *Cytometry*, **2**, 238.

STRAUSS, G.H. & ALBERTINI, R.J. (1979). Enumeration of 6-thioguanine-resistant peripheral blood lymphocytes in man as a potential test for somatic cell mutations arising *in vivo*. *Mutat. Res.*, **61**, 353.

VOLLER, A., BARTLETT, A. & BIDEWELL, D.E. (1978). Enzyme immunoassays with special reference to ELISA techniques. *J. Clin. Pathol.*, **31**, 507.

WADDELL, D, & ULLMAN, B. (1983). Characterization of a cultured human T-cell line with genetically altered ribonucleotide reductase activity. *J. Biol. Chem.*, **258**, 4226.

WARREN, S.T., SCHULTZ, R.A., CHANG, C.C., WADE, M.H. & TROSKO, J.E. (1981). Elevated spontaneous mutation rate in Bloom syndrome fibroblasts. *Proc. Natl Acad. Sci.* (*USA*), **78**, 3133.


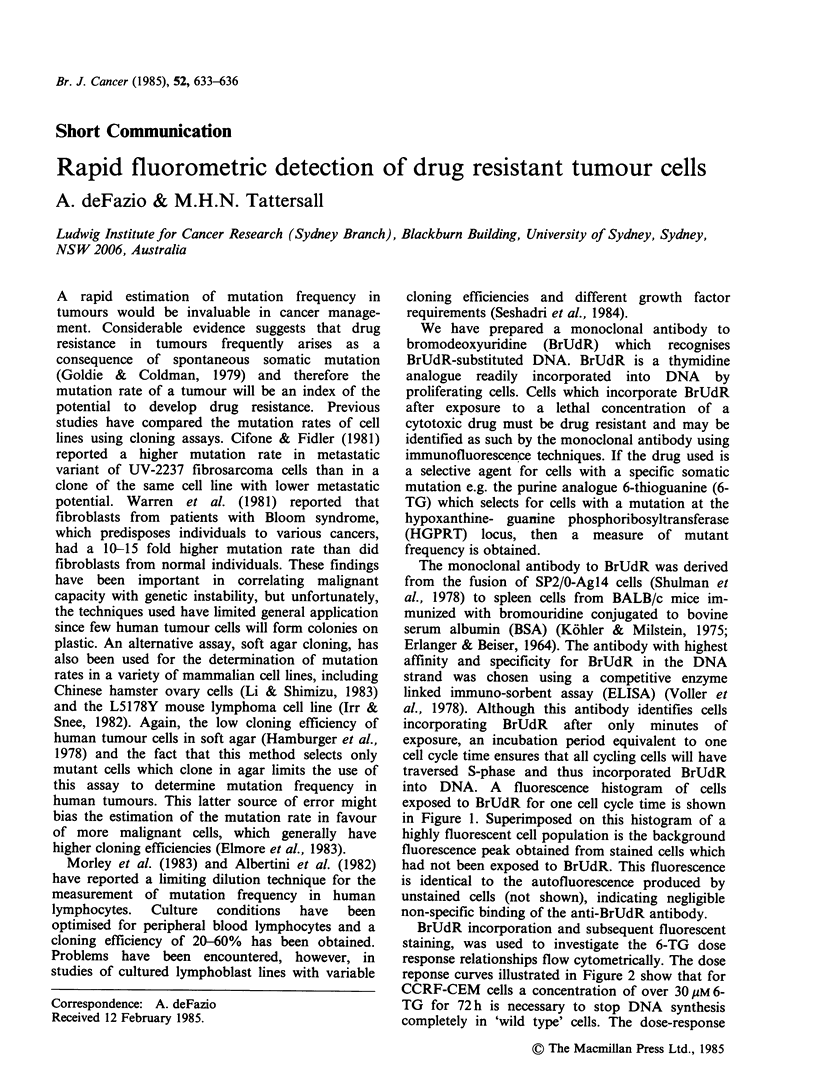

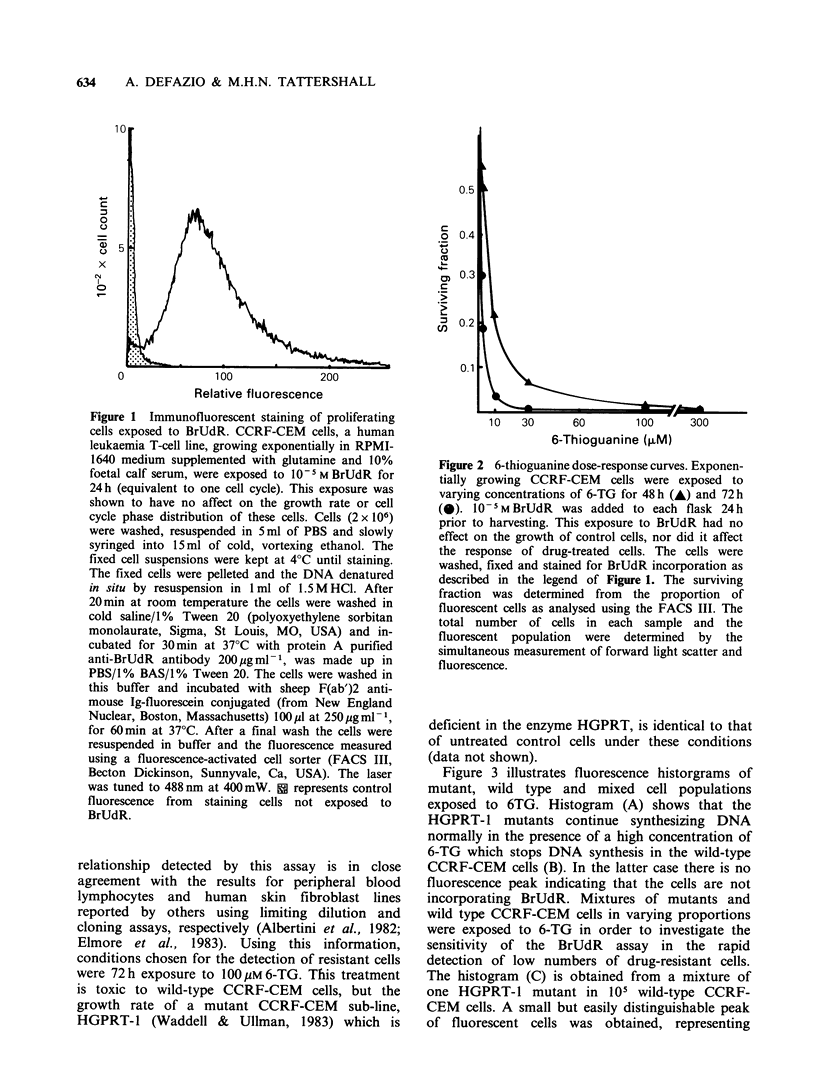

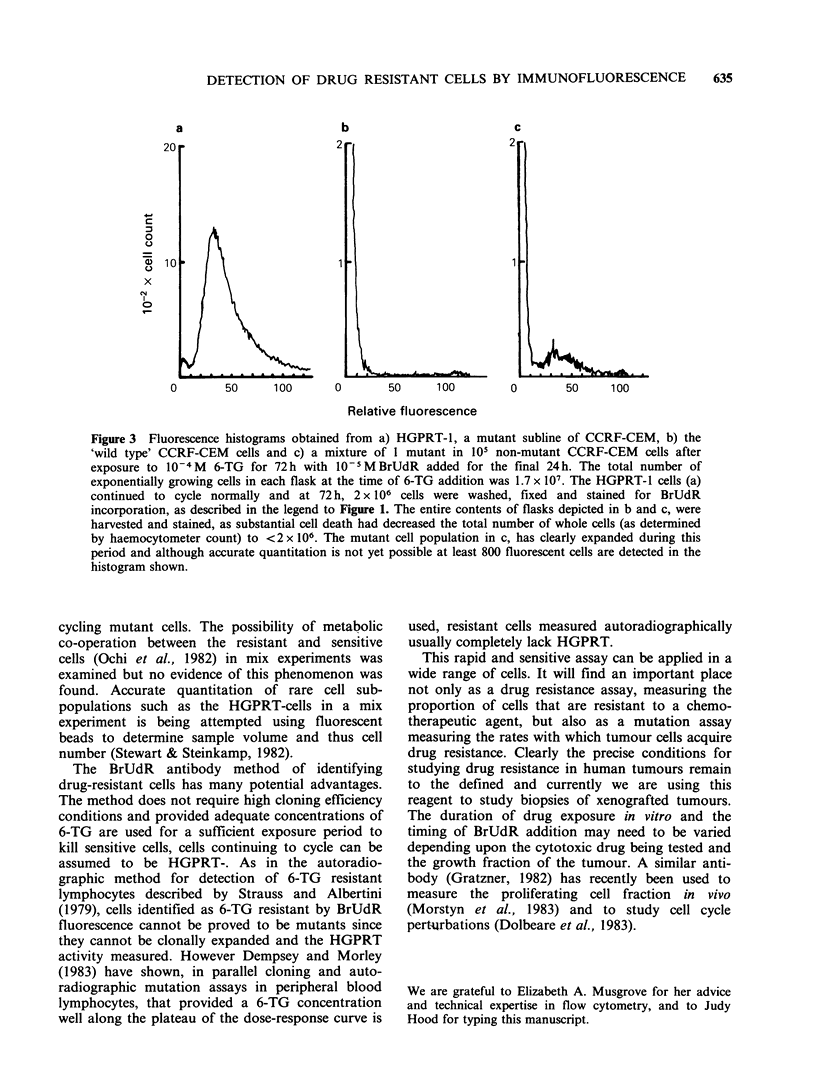

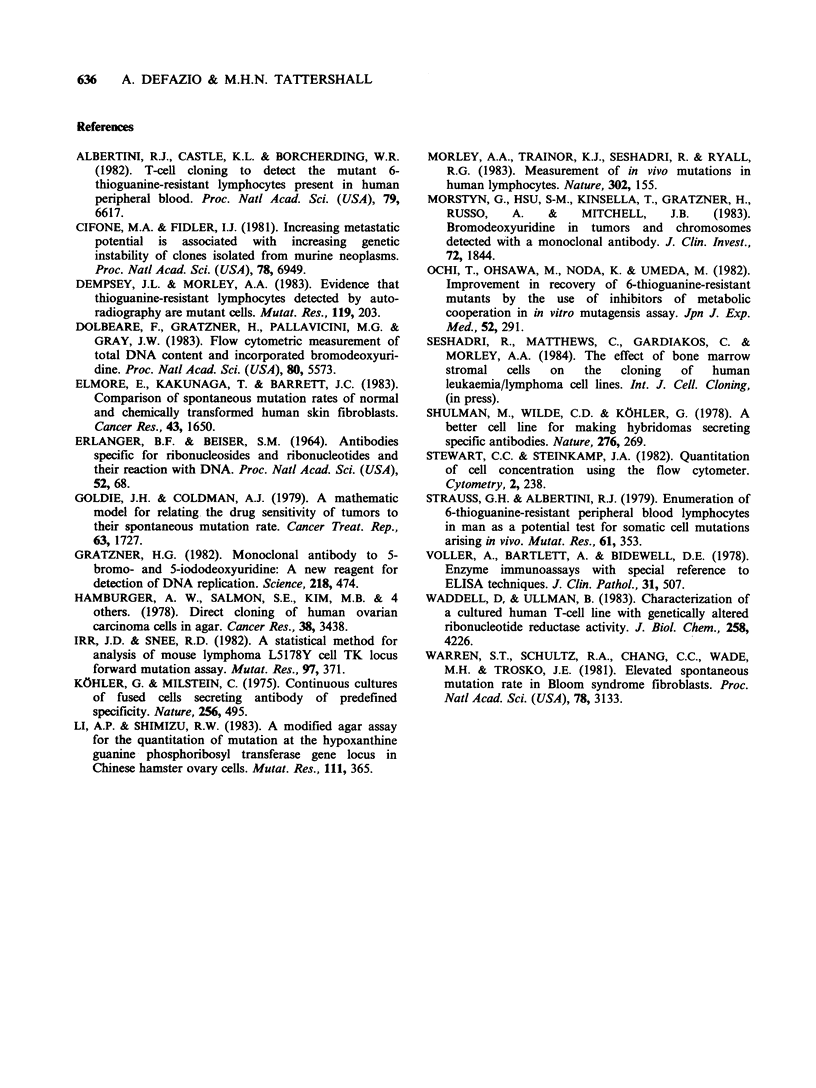


## References

[OCR_00330] Albertini R. J., Castle K. L., Borcherding W. R. (1982). T-cell cloning to detect the mutant 6-thioguanine-resistant lymphocytes present in human peripheral blood.. Proc Natl Acad Sci U S A.

[OCR_00337] Cifone M. A., Fidler I. J. (1981). Increasing metastatic potential is associated with increasing genetic instability of clones isolated from murine neoplasms.. Proc Natl Acad Sci U S A.

[OCR_00343] Dempsey J. L., Morley A. A. (1983). Evidence that thioguanine-resistant lymphocytes detected by autoradiography are mutant cells.. Mutat Res.

[OCR_00348] Dolbeare F., Gratzner H., Pallavicini M. G., Gray J. W. (1983). Flow cytometric measurement of total DNA content and incorporated bromodeoxyuridine.. Proc Natl Acad Sci U S A.

[OCR_00360] ERLANGER B. F., BEISER S. M. (1964). ANTIBODIES SPECIFIC FOR RIBONUCLEOSIDES AND RIBONUCLEOTIDES AND THEIR REACTION WITH DNA.. Proc Natl Acad Sci U S A.

[OCR_00354] Elmore E., Kakunaga T., Barrett J. C. (1983). Comparison of spontaneous mutation rates of normal and chemically transformed human skin fibroblasts.. Cancer Res.

[OCR_00366] Goldie J. H., Coldman A. J. (1979). A mathematic model for relating the drug sensitivity of tumors to their spontaneous mutation rate.. Cancer Treat Rep.

[OCR_00372] Gratzner H. G. (1982). Monoclonal antibody to 5-bromo- and 5-iododeoxyuridine: A new reagent for detection of DNA replication.. Science.

[OCR_00377] Hamburger A. W., Salmon S. E., Kim M. B., Trent J. M., Soehnlen B. J., Alberts D. S., Schmidt H. J. (1978). Direct cloning of human ovarian carcinoma cells in agar.. Cancer Res.

[OCR_00382] Irr J. D., Snee R. D. (1982). A statistical method for analysis of mouse lymphoma L5178Y cell TK locus forward mutation assay. Comparison of results among three laboratories.. Mutat Res.

[OCR_00387] Köhler G., Milstein C. (1975). Continuous cultures of fused cells secreting antibody of predefined specificity.. Nature.

[OCR_00392] Li A. P., Shimizu R. W. (1983). A modified agar assay for the quantitation of mutation at the hypoxanthine guanine phosphoribosyl transferase gene locus in Chinese hamster ovary cells.. Mutat Res.

[OCR_00398] Morley A. A., Trainor K. J., Seshadri R., Ryall R. G. (1983). Measurement of in vivo mutations in human lymphocytes.. Nature.

[OCR_00403] Morstyn G., Hsu S. M., Kinsella T., Gratzner H., Russo A., Mitchell J. B. (1983). Bromodeoxyuridine in tumors and chromosomes detected with a monoclonal antibody.. J Clin Invest.

[OCR_00410] Ochi T., Ohsawa M., Noda K., Umeda M. (1982). Improvement in recovery of 6-thioguanine-resistant mutants by the use of inhibitors of metabolic cooperation in in vitro mutagenesis assay.. Jpn J Exp Med.

[OCR_00424] Shulman M., Wilde C. D., Köhler G. (1978). A better cell line for making hybridomas secreting specific antibodies.. Nature.

[OCR_00429] Stewart C. C., Steinkamp J. A. (1982). Quantitation of cell concentration using the flow cytometer.. Cytometry.

[OCR_00434] Strauss G. H., Albertini R. J. (1979). Enumeration of 6-thioguanine-resistant peripheral blood lymphocytes in man as a potential test for somatic cell mutations arising in vivo.. Mutat Res.

[OCR_00440] Voller A., Bartlett A., Bidwell D. E. (1978). Enzyme immunoassays with special reference to ELISA techniques.. J Clin Pathol.

[OCR_00445] Waddell D., Ullman B. (1983). Characterization of a cultured human T-cell line with genetically altered ribonucleotide reductase activity. Model for immunodeficiency.. J Biol Chem.

[OCR_00451] Warren S. T., Schultz R. A., Chang C. C., Wade M. H., Trosko J. E. (1981). Elevated spontaneous mutation rate in Bloom syndrome fibroblasts.. Proc Natl Acad Sci U S A.

